# Individual strategy ratings improve the control for task difficulty effects in arithmetic problem solving paradigms

**DOI:** 10.3389/fpsyg.2015.01188

**Published:** 2015-08-13

**Authors:** Nadja Tschentscher, Olaf Hauk

**Affiliations:** Cognition and Brain Sciences Unit, Medical Research CouncilCambridge, UK

**Keywords:** problem solving, arithmetic cognition, task complexity, Receiver Operating Characteristic, neuroimaging

## Abstract

Mental arithmetic is a powerful paradigm to study problem solving using neuroimaging methods. However, the evaluation of task complexity varies significantly across neuroimaging studies. Most studies have parameterized task complexity by objective features such as the number size. Only a few studies used subjective rating procedures. In fMRI, we provided evidence that strategy self-reports control better for task complexity across arithmetic conditions than objective features (Tschentscher and Hauk, 2014). Here, we analyzed the relative predictive value of self-reported strategies and objective features for performance in addition and multiplication tasks, by using a paradigm designed for neuroimaging research. We found a superiority of strategy ratings as predictor of performance above objective features. In a Principal Component Analysis on reaction times, the first component explained over 90 percent of variance and factor loadings reflected percentages of self-reported strategies well. In multiple regression analyses on reaction times, self-reported strategies performed equally well or better than objective features, depending on the operation type. A Receiver Operating Characteristic (ROC) analysis confirmed this result. Reaction times classified task complexity better when defined by individual ratings. This suggests that participants’ strategy ratings are reliable predictors of arithmetic complexity and should be taken into account in neuroimaging research.

## Introduction

The difficulty of any given problem is a function of individual problem solving skills and task features. Hence, task complexity in an experimental setting can be either defined by participants’ problem solving strategies and performance measures, or *a priori*, by considering a discrete number of objective task features (Imbo et al., 2007). Mental arithmetic has proven to be a powerful paradigm to study various aspects of abstract problem solving in neuroimaging experiments (Zhou et al., 2007; Jost et al., 2009; Rosenberg-Lee et al., 2009; Arsalidou and Taylor, 2011), due to the simplicity of numerical stimuli and clear formal structure of arithmetic problems.

However, it is still an open question which criterion may predict task complexity best across differential problem solving conditions. While *a priori* defined task features for complexity have the advantage of being based on explicit and objective definitions, the complexity of a task for an individual participant on an individual trial may be better captured by assessment of their individual strategies. In order to evaluate these approaches, one needs independent criteria for the success of a complexity criterion. The ideal choice would depend on a detailed knowledge of the cognitive mechanisms contributing to task complexity, how they vary across individuals, and how they are affected by experimentally controlled stimulus and task parameters. In many studies, this is far from achievable. We here provide a pragmatic solution with an arithmetic task paradigm that allows assessment of performance and individual participants’ strategies in neuroimaging research. For this paradigm, we determined the relative explanatory value of objective task features (e.g., problem size) and individual strategy ratings (e.g., “In how many steps did you solve this problem?”) on performance.

Previous neuroimaging research in the domain of arithmetic cognition has mainly focused on objective task features when defining levels of task complexity, while not much attention has been paid to assessment of participants’ problem solving strategies. Most studies defined task complexity based on number size (cf. Jost et al., 2004, 2009; Rosenberg-Lee et al., 2011), the number of involved operands (Menon et al., 2000), or carry-effects (whether the solution exceeds the next 10s) (Kong et al., 2005). Overall, number size has been the most used criterion of task complexity in neuroimaging research. It has been argued that number size may reflect the differential use of strategies involving direct memory retrieval of answers vs. solution of problems in several sub-steps (cf. Jost et al., 2009; Arsalidou and Taylor, 2011). However, previous behavioral studies already demonstrated that even tasks involving only two single digits, for which solutions are often assumed to be retrieved from memory, may be solved by procedural strategies (LeFevre et al., 1996a,b, 2006; Barrouillet and Thevenot, 2013), and the amount of tasks solved by direct memory retrieval is determined by participants’ overall skill level (Hecht, 2006).

Objective task complexity measures do not account for individual differences, and are particularly problematic when aiming to evaluate neural differences between arithmetic operation types (for example, addition, subtraction, and multiplication) independently of task complexity effects. It has been, for example, suggested that addition and multiplication genuinely differ regarding the application of cognitive strategies used to solve each of these operation types. Addition may more strongly involve visual-spatial and sensorimotor processes while multiplication may more strongly rely on direct memory retrieval (Lakoff and Núñez, 2000; Fischer, 2012; Hauk and Tschentscher, 2013). This has been shown by behavioral (Badets et al., 2010; Klein et al., 2011) as well as neuroimaging research (Zhou et al., 2006, 2007; Grabner et al., 2009; Rosenberg-Lee et al., 2011). However, these studies mostly defined complexity based on task features, yielding a mismatch across operation types in accuracy and reaction times (Chochon et al., 1999; Zhou et al., 2006; Grabner et al., 2009; Rosenberg-Lee et al., 2011). Thus, results from these studies may have been confounded by task complexity effects.

So far, only very few neuroimaging studies assessed task complexity via individual problem solving strategies (De Smedt et al., 2009; Grabner et al., 2009; Grabner and De Smedt, 2011; Tschentscher and Hauk, 2014). A recent fMRI study suggested that trial-by-trial strategy self-reports provide a more accurate measure of task complexity across different operation types, and previously reported neural differences between addition and multiplication problems vanished once task complexity was defined by strategy ratings (Tschentscher and Hauk, 2014). Self-reported strategies also revealed more sensitive measures of task complexity in EEG recordings compared to complexity definitions based on number size (Grabner and De Smedt, 2011), and effects of training in oscillatory EEG correlates were reflected in increases of reported retrieval strategies as well as decreased error rates and reaction times (Grabner and De Smedt, 2012). Therefore, the assessment of individual strategies may provide a more reliable measure of complexity across different types of problems. This is in line with several behavioral studies showing a high consistency between strategy reports and mean reaction times (LeFevre et al., 1996a; Baroody and Dowker, 2003), as well as parameters from modeling of reaction times (Campbell and Penner-Wilger, 2006).

In the present study, we asked participants to solve addition and multiplication tasks that consisted of two operands. While production tasks with verbal responses have been used in most behavioral studies on mental arithmetic (cf. LeFevre et al., 2003), our design required the pressing of a button as soon as participants knew the answer, which then triggered the onset of a 2-Alternative-Forced-Choice (2AFC) result display. The result display remained on screen for very short time only, thus discouraging participants to “pre-emptively” press the button before they finished calculating. A similar design has been used in combination with a voice-key before, in which a button was pressed before answers were verbally indicated (Lemaire and Arnaud, 2008). Our paradigm provides reliable reaction time measures even in a noisy environment, such as in the MRI scanner, and has the advantage that the answer options are still unknown during the calculation phase, as opposed to a classical 2AFC paradigm.

We assessed task complexity on a trial-by-trial basis using strategy ratings (i.e., reports of direct memory retrieval, 1-step calculation, 2-steps, 3-steps, or more steps). Such a rating scale has been used in three previous neuroimaging studies (Grabner and De Smedt, 2011, 2012; Tschentscher and Hauk, 2014), and allows a task complexity assessment with comparable results across participants, once subjects are instructed and trained in appropriate use of the scale. We investigated whether number size based task features or strategy ratings predict behavioral performance best across a range of addition and multiplication tasks. We *a priori* grouped together arithmetic problems that may involve similar strategies, based on the idea that certain types of tasks are frequently solved by a particular “shortcut” which simplifies the problem solving process, compared to the solution of other tasks with equivalent or even smaller operands. For example, tasks involving the multiplier 9 (“9 × 6”) might be solved faster than tasks involving the multipliers 7 or 8 (i.e., “7 × 6,” or “8 × 6”) since a “shortcut” strategy could be applied: 9 × 6 = [10 × 6] – 6. Hence, the task feature of “number size” may in this case not be a reliable predictor of task complexity. Whether or not participants make use of such shortcut strategies may depend on individual skill levels, and cannot be inferred from task features.

A Principal Component Analysis (PCA) was applied first to our reaction time data to determine whether performance across categories of problems can be described by a single dimension, presumably reflecting problem difficulty. We then applied a multiple linear regression analysis in order to decide whether predictors derived from objective task features or individual ratings best explain performance. Finally, we used a Receiver Operator Characteristic (ROC) analysis to test whether reaction times classified task complexity better when it was based on individual ratings than when it was based on objective task features.

## Materials and Methods

### Participants

Data from 21 adult participants (10 males; 11 females) were obtained. All participants were right-handed, had normal or corrected-to-normal vision, and had no history of neurological or psychiatric disorder. Participants’ IQ was assessed by using the Culture-Fair-Test, Scale 2 (Cattell and Cattell, 1960). The mean age of the analyzed participants was 22 years (SD: 3.25), and the mean IQ was 127.84 (SD: 14.61). Participants were recruited from the MRC Cognition and Brain Sciences Unit’s internal volunteer panel, and received about £10 for their participation. Ethical approval was obtained from the Cambridge Local Research Ethics Committee.

### Stimuli

Based on number sizes, five different levels of task complexity were initially defined for addition tasks, and three different levels of task complexity were defined for multiplication tasks (**Table 1**). These were chosen based on complexity levels reported in previous literature (Fehr et al., 2007; Grabner et al., 2009; Arsalidou and Taylor, 2011; Tschentscher and Hauk, 2014). We also included task categories with highly frequent problems, for example ties (“6 + 6” or “7 × 7”) or problems involving the multipliers 2 (“2 × 3” or “2 × 4”). Further, task categories were defined that might be solved by a shortcut strategy. These were problems involving the multipliers 5 or 9. In the case of tasks involving the multiplier 5, a typical “shortcut-strategy” could be for example: 5 × 6 = [10 × 6]/2. In the case of tasks with the multiplier 9, a shortcut strategy might look as follows: 9 × 6 = [10 × 6] – 6. This implies that the problem of for example “9 × 3” would be solved faster than the problem “8 × 3,” although the sum of operands is larger in the first case, compared to the latter case. Hence, surface criteria such as number size may not reflect task complexity accurately for these problem categories, and it was expected that participants would mainly indicate the use of memory retrieval or easy procedural strategies.

**Table 1:** Task categories based on surface criteria.

Addition		Multiplication	
Number size based complexity levels	Other surface criteria “shortcuts”	Number size based complexity levels	Other surface criteria “shortcuts”
Level 1: 1–9 vs. 1–9 Number of Trials: 20 IA = ±2	Ties: e.g., 9 + 9 Number of Trials: 8 IA =±2	Level 1: 1–9 vs. 1–9 Number of Trials: 20 IA = ±2	Ties: e.g., 6 × 6 Number of Trials: 8 IA = ±2
Level 2: 1–9 vs. 12–19 Number of Trials: 20 IA = ±2	Sum to ten (a): e.g., 8 + 2 Number of Trials: 4 IA = ±2	Level 2: 1–9 vs. 12–19 Number of Trials: 20 %50 IA = ±2 %50 IA = ±10	Multiplier 2 easy: e.g., 2 × 4 Number of Trials: 10 IA = ±2
Level 3: 12–19 vs. 12–19 Number of Trials: 20 IA = ±2	Sum to ten (b): e.g., 14 + 6 Number of Trials: 8 IA = ±2	Level 3: 12–19 vs. 12–19 Number of Trials: 20 %50 IA = ±2 %50 IA = ±10	Multiplier 2 hard: e.g., 2 × 19 Number of Trials: 10 IA = ±2
Level 4: 12–19 vs. 21–59 Number of Trials: 20 %50 IA = ±2 %50 IA = ±10	Sum to ten (c): e.g., 13 + 17 Number of Trials: 12 IA = ±2		Multiplier 5 easy: e.g., 3 × 5 Number of Trials: 10 IA = ±5
	Sum to ten with 5: e.g., 5 + 15 Number of Trials: 20 IA = ±5		Multiplier 5 hard: e.g., 5 × 17 Number of Trials: 10 IA = ±5
			Multiplier 9 easy: e.g., 9 × 7 Number of Trials: 10 IA = ±2
Level 5: 21–59 vs. 21–59 Number of Trials: 20 %50 IA = ±2 %50 IA = ±10			Multiplier 9 hard: e.g., 16 × 9 Number of Trials: 10 IA = ±2

*The range of incorrect answers (IAs) is given for each category.*

Twenty problems were presented for each number size based subcategory, all matched according to the following criteria: odd and even numbers, the order of presenting the larger operand, carry-over effects (in which the solution exceeds the next 10), and multipliers larger vs. smaller than five in multiplication problems. Participants had to choose between two result options (correct and incorrect) in a 2AFC task. Incorrect options (IA, see **Table 1**) were matched in surface features with the correct solutions (i.e., regarding parity, and the multiplicative of 5, where appropriate), and varied between plus/minus 2, 5, or 10, depending on the correct solution. Overall, 152 addition tasks and 138 multiplication tasks were presented in the experiment (see **Table 1** for details on trial numbers within each subcategory).

### Procedure

**FIGURE 1:**
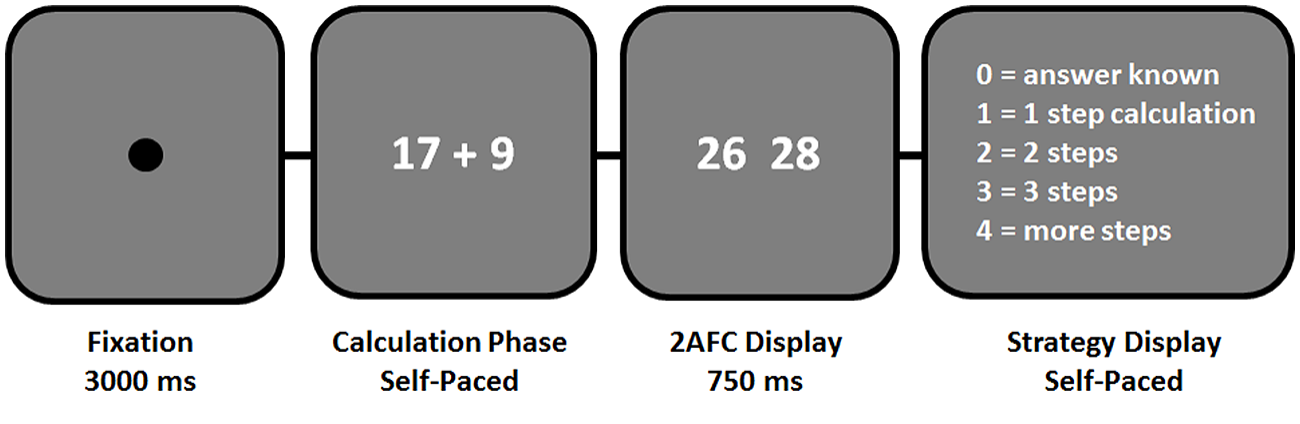
**Illustration of trial structure used in the experiment.** Participants were instructed to work out the solution as fast and accurately as possible, and to press a button as soon as they knew the answer, which triggered the onset of a 2-Alternative-Forced-Choice (2AFC) result display. The result display remained on screen for 750 ms only, in order to discourage participants to pre-emptively press the button before they finished calculating. Arithmetic strategies were assessed on a trial-by-trial basis by ratings on the number of solution steps.

Problems were presented on a computer screen in a conventional format “operand–operator–operand” and participants were instructed to work out the solution as fast and accurately as possible. They were told to press a button as soon as they knew the answer, which triggered the onset of a 2AFC result display (**Figure 1**). The result display remained on screen for 750 ms. This discouraged participants to “pre-emptively” press the button before they finished calculating. Subsequently, participants had to indicate their arithmetic strategy by rating the number of solution steps. The following rating options were given: answer known (i.e., direct memory retrieval), 1-step calculation (e.g., 10 + 15 = 20 + 5), 2-steps (e.g., 14 + 17 = 20 + 4 + 7), 3-steps (e.g., 27 + 36 = 20 + 30 + 7 + 6), or more steps. Examples for each rating option were given in a 15-trial practice session. Tasks were randomized within eight blocks, containing around 30 trials per block. The inter-stimulus-interval was 3 s. Fluid intelligence measures from the Culture Fair Intelligence Test (Cattell and Cattell, 1960) were obtained after the behavioral experiment.

### Statistical Analyses

First, a PCA was conducted on reaction times across participants and task categories (see **Table 1**). The reaction times for each task category were averaged for individual subjects, and then entered into a PCA across subjects for addition and multiplication tasks separately. This was done in order to explore (a) whether categories correlated highly with each other and could be subsumed under one category, and (b) whether the component loadings reflected specific self-reported strategies. For this, mean reaction times and mean percentages of strategy rating options were compared with results of the PCA analysis.

Second, the relative impact of individual strategy ratings compared to task surface criteria was assessed in a multiple regression analysis. Independent simultaneous multiple linear regression analyses were run on reaction times of individual subjects including the predictor “Strategy” (retrieval, 1–3 steps, and more steps), and either of the two number size predictors “Sum of Operands” and “Product of Operands.” Separate multiple regression analyses were run for all tasks (addition and multiplication together), as well as for addition and multiplication tasks. The mean percentage of partially explained variance by each predictor was determined for each of the described regression models across subjects. The relative predictive value of strategy and number size predictors was analyzed by subjecting *z*-transformed values of explained variance to paired-sample *t*-tests.

Third, in order to investigate whether reaction times determine strategy type or task features more appropriately, we ran a ROC analysis. This method from signal detection theory classifies a data set into two groups based on a variable of interest (Centor, 1991). For any given threshold, the proportion of cases that are correctly classified into a particular group is determined (i.e., the true positives), as well as the percentage of cases that are incorrectly classified into the same group (false positives). The threshold is varied to yield an ROC curve that indicates the point, at which the proportion of true positives and false positives is optimal, i.e., true positives are maximized while false positives are minimized. The Area under the Curve (AUC) provides a measure of the likelihood at which the ROC curve correctly classifies between true positives and true negatives (Fawcett, 2006). An AUC of 0.50 indicates chance level, while an AUC of 1 indicates perfect classification. In recent years, a few studies used ROC analyses to determine whether reaction times accurately classify arithmetic strategy choices of primary school children with different skill levels (Mazzocco and Thompson, 2005; Wu et al., 2008). We here focus on the relative classification accuracy of strategy ratings and task features across a heterogeneous group of participants, for a broad range of task conditions. ROC analyses were conducted for ratings (retrieval vs. any procedural strategy) and task features (1-digit vs. multiple-digits tasks) of all arithmetic tasks, as well as separately for addition and multiplication tasks. The ROC function provided by the Matlab (R2009a) software was used. For statistical analyses, AUC values were *z*-transformed, and tested for normality (Lilliefors, 1969). In order to investigate differences in classification accuracy of ratings and task features, paired-sample *t*-tests were applied to AUC values of all tasks, as well as to AUC values of addition and multiplication tasks.

## Results

Data of 19 participants (11 females and 8 males) were analyzed. Two participants were excluded from the initial group because they did not use the rating scale appropriately, i.e., did not report any retrieval strategies whatsoever, and chose one option on the rating scale over-proportionally frequently, despite the fact that their reaction times were within the mean and standard deviation of all other participants. This suggests that these participants failed to follow our instructions. The mean error rate across all task categories was 11 percent (SD = 6.07). Reaction times for error-trials were excluded from analyses, as well as outliers of three standard deviations above the mean of addition and multiplication tasks.

### Principal Component Analysis

**FIGURE 2:**
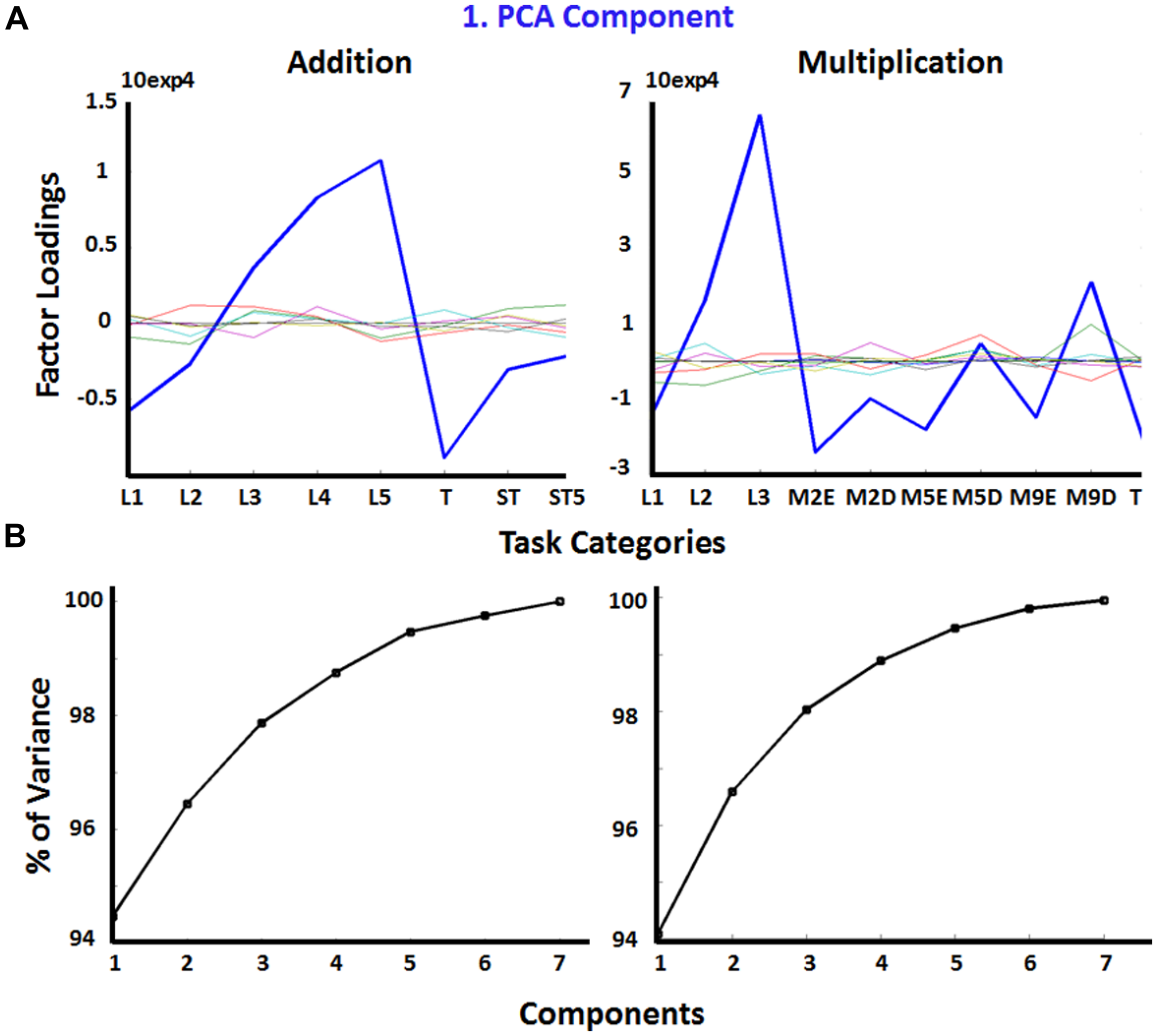
**(A)** Factor loadings of PCA components for addition and multiplication tasks across pre-defined task categories. The blue line indicates the first PCA component out of the 7 components (in different colors) that together explained approximately a hundred percent of variance (see **B**). **(B)** Cumulative variances for PCA components for additions (left) and multiplication (right). *x*-Axis labels for addition in **(A)** (see **Table 1**): L1–5, number size based complexity levels; T, ties; ST, sum to next 10s; ST5, sum to next tens by adding 5. *x*-Axis labels for multiplication in **(A)** (see **Table 1**): L1–3, number size based complexity levels; M2E, multiply by 2 (easy); M2D, multiply by 2 (difficult); M5E, multiply by 5 (easy); M5D, multiply by 5 (difficult); M9E, multiply by 9 (easy); M9D, multiply by 9 (difficult); T, ties.

A PCA on reaction times was run in order to explore to what degree pre-defined task categories (e.g., shortcut strategies) differentially affect performance. The first component accounted for 94 percent of variance in reaction times of both operation types (see **Figure 2** for overview of factor loadings of all PCA components, and for cumulated percentages of variance explained by each of the components). The first two components explained 96 percent of variance in reaction times of addition and multiplication tasks. Six components for addition tasks and eight components for multiplication tasks explained ∼100 percent of variance in the data (see **Table 2** for component loadings).

**Table 2:** Component loading matrix of PCA analysis, corresponding to **Figure 3**.

	Component Loading Matrix					
	**Addition**					
	**C1**	**C2**	**C3**	**C4**	**C5**	**C6**	

Level 1	-5789.40	-910.53	-6.58	297.57	-125.05	652.58		
Level 2	-2750.19	-1084.20	1199.93	-847.91	-182.14	-243.05		
Level 3	3537.96	1065.17	641.97	710.55	-906.43	37.42		
Level 4	8184.92	316.74	716.60	240.43	844.13	-121.15		
Level 5	10496.82	-1140.74	-1087.75	-33.26	-166.02	4.66		
Ties	-8679.49	-272.48	-516.70	859.28	226.27	-548.55		
Sum-to-next-ten	-3000.45	952.40	-163.39	-262.70	601.81	404.99		
Sum-to-next-ten by adding 5	-2000.15	1073.65	-784.08	-963.97	-292.56	-186.91		

	**Multiplication**							
	**C1**	**C2**	**C3**	**C4**	**C5**	**C6**	**C7**	**C8**

Level 1	-15226.81	-5528.17	-3162.14	186.70	-3104.47	2433.10	496.86	-25.36
Level 2	15713.04	-6431.04	-2286.43	4629.61	2120.84	-1596.28	89.04	-2.66
Level 3	63569.99	-2545.53	1999.25	-3436.67	-965.49	-254.24	-71.92	16.84
Multiply 2 eay	-23466.06	1453.94	1942.28	-1587.48	-367.60	-2166.47	672.85	-518.03
Multiply 2 difficult	-10113.76	644.65	-1909.13	-3034.98	4604.08	1491.68	251.31	287.12
Multiply 5 eay	-17882.24	358.63	1675.46	165.66	-1024.35	-645.26	-1858.88	846.75
Multiply 5 difficult	4547.63	3036.39	6959.88	3359.05	564.39	1920.56	321.94	-160.37
Multiply 9 eay	-14859.47	-280.95	-1061.56	-911.23	162.68	134.72	-1440.19	-924.68
Multiply 9 difficult	20543.67	9681.05	-5197.44	1594.01	-1041.58	-133.95	186.60	56.00
Tie	-22825.97	-388.95	1039.83	-964.66	-948.48	-1183.85	1352.37	424.39

*The six components (C1–C6) for addition tasks are presented, as well as the eight components (C1–C8) for multiplication tasks, which together explained ∼100 percent of variance.*

**FIGURE 3:**
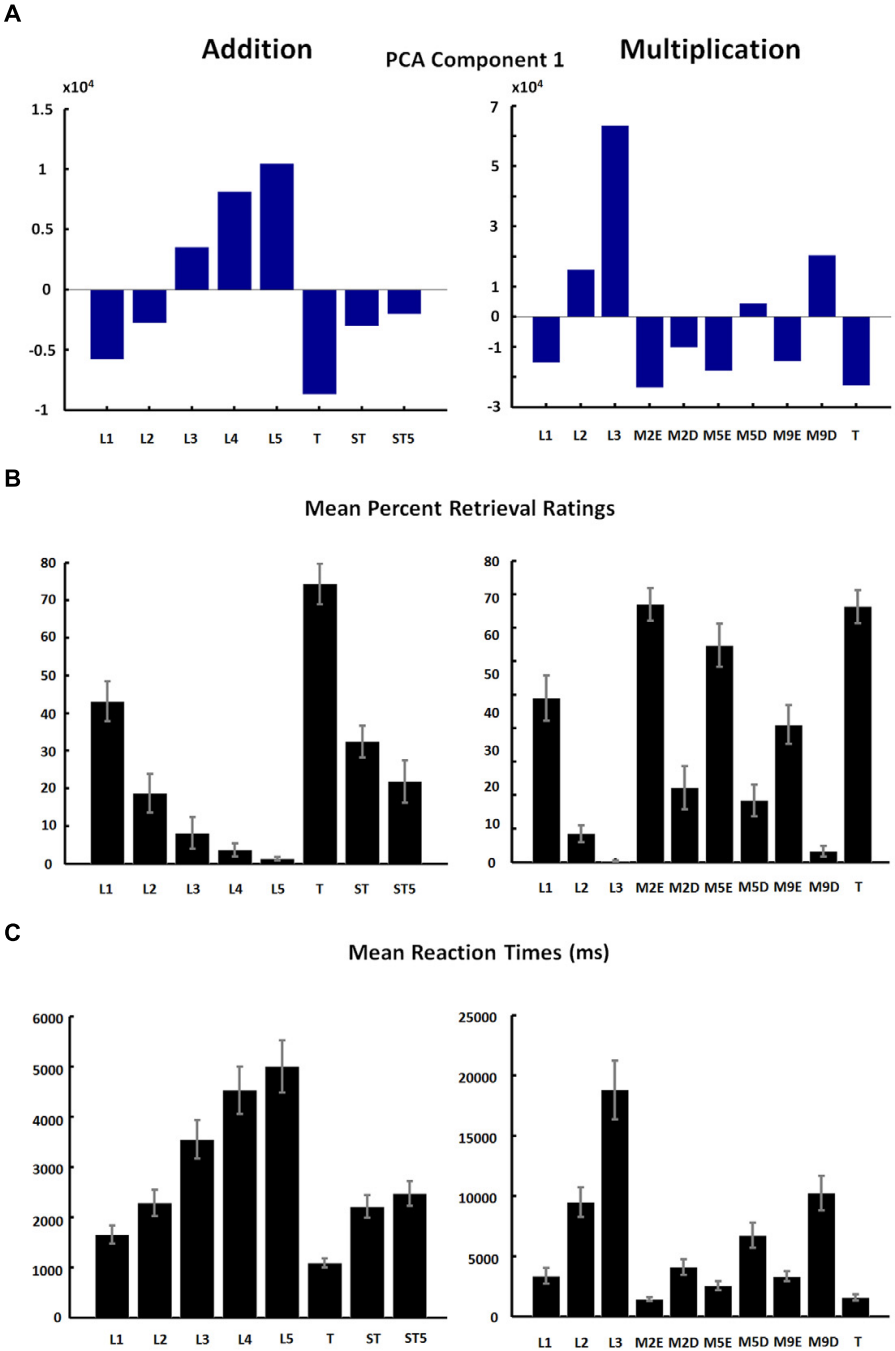
**(A)** Factor loadings of first PCA component (same as in **Figure 2A**); **(B)** mean percentage of retrieval ratings of each pre-defined category; **(C)** mean reaction times in pre-defined categories presented. Error bars indicate the SE of the mean. Abbreviations: L1–L5, number size based complexity levels; T, ties; ST, sum to next 10s; ST5, sum to next 10s by adding 5; M2E, multiply by 2 (easy); M2D, multiply by 2 (difficult); M5E, multiply by 5 (easy); M5D, multiply by 5 (difficult); M9E, multiply by 9 (easy); M9D, multiply by 9 (difficult).

The loadings of the first component (**Figure 3A**, see **Table 2** for Component Loading Matrix) closely matched the mean percentages of retrieval ratings (**Figure 3B**), as well as the pattern of mean reaction times (**Figure 3C**) across task categories. This suggests that the amount as to which subjects use the direct memory retrieval strategy, as opposed to any procedural strategy, predicts reaction times well across trials, and explains most variance in the PCA analysis. This was the case for addition as well as multiplication tasks. Interestingly, high factor loadings of the first component emerged for task categories including a wide range of number sizes, ranging from 1/1-digit tasks to 2/2-digits tasks. This suggests that arithmetic task complexity is not well-explained by task surface features, but may rather reflect participants’ strategy ratings.

### Multiple Regression Analysis

In a multiple regression analysis, we determined the relative predictive value of strategy ratings and task features. The predictor “Strategy” included the five rating options (retrieval, 1–3 steps, and more steps), and was evaluated together with the number size predictors “Sum of Operands” as well as “Product of Operands.” Simultaneous multiple linear regressions were run on reaction times of all arithmetic tasks, as well as on reaction times of addition and multiplication tasks. The inter-correlation of strategy and number size predictors was around 0.6, and did not vary much across regression analyses of all tasks, as well as addition and multiplication (**Table 3B**). Collinearity statistics showed a mean Variance Inflation Factor (VIF) between 1.4 and 2.45 across the different regression models (see **Table 3C** for details). Due to these relatively high inter-correlations, the absolute values of regression coefficients should be interpreted with caution. Hence, we here only analyzed the relative differences of partially explained variance between predictors. The relative impact of strategy and number size predictors was analyzed by subjecting *z*-transformed values of explained variance to paired-sample *t*-tests.

**Table 3:** **(A)** Mean percent and SD of partially explained variance by each predictor from multiple regressions on reaction times run on the single-subject level for all tasks, as well as addition and multiplication tasks. **(B)** Mean and SD of predictor correlations from multiple regressions on the single-subject level run for all tasks, as well as addition and multiplication tasks. **(C)** Mean and SD of the Variance Inflation Factor (VIF) from multiple regressions on the single-subject level run for all tasks, as well as addition and multiplication tasks.

(A) Partial *R*^2^ Coefficients				
	**Strategy**		**Sum of operands**	
	**Mean**	**SD**	**Mean**	**SD**

All Tasks	0.13	0.08	0.09	0.04
Addition	0.23	0.12	0.10	0.08
Multiplication	0.22	0.15	0.17	0.09

	**Strategy**		**Product of operands**	
	**Mean**	**SD**	**Mean**	**SD**

All Tasks	0.16	0.08	0.11	0.04
Addition	0.29	0.12	0.09	0.08
Multiplication	0.25	0.14	0.27	0.10

**(B) Correlations of Predictors**				

	**Sum of operands ^∗^ Strategy**		**Product of operands ^∗^ Strategy**	
	**Mean**	**SD**	**Mean**	**SD**

All tasks	0.66	0.07	0.59	0.06
Addition	0.62	0.07	0.53	0.07
Multiplication	0.74	0.07	0.68	0.09

**(C) Variance Inflation Factor (VIF)**				

	**Sum of operands vs. Strategy**		**Product of operands vs. Strategy**	
	**Mean**	**SD**	**Mean**	**SD**

All Tasks	1.90	0.31	1.58	0.18
Addition	1.70	0.26	1.41	0.16
Multiplication	2.45	0.75	2.03	0.49

**FIGURE 4:**
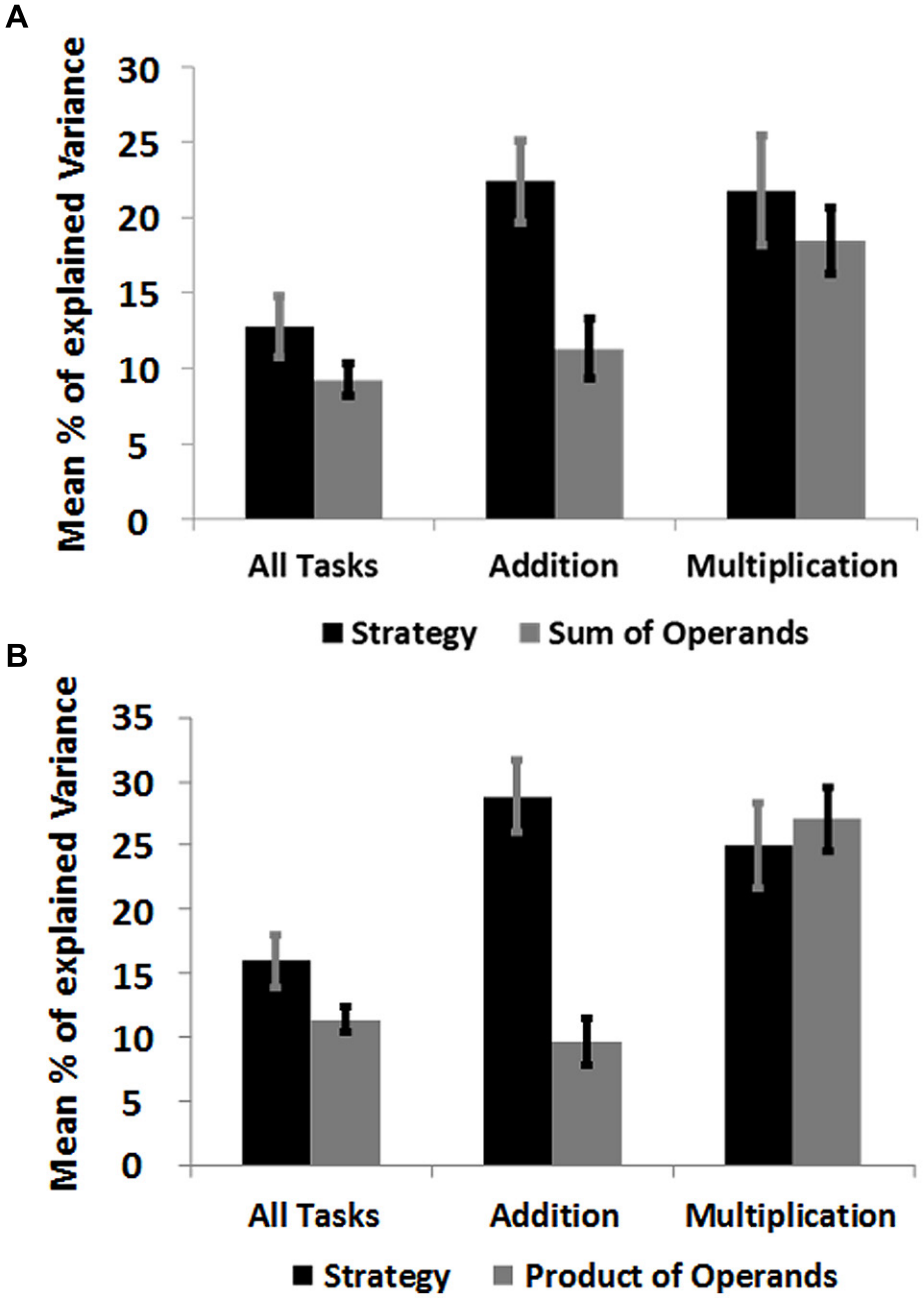
**(A)** Mean explained variance by each predictor in multiple regression of reaction times on the standardized predictors “Strategy” (retrieval, 1-step, 2-steps, 3-steps, more-steps) and “Sum of Operands” for all task, as well as addition and multiplication. Error bars indicate the SE of the mean. **(B)** Mean explained variance by each predictor in multiple regressions of reaction times on the standardized predictors “Strategy” and “Product of Operands” for all task, as well as addition and multiplication.

Strategy ratings explained most of the variance in reaction times of addition tasks compared to number size predictors (**Figures 4A,B**; **Table 3A**). This was the case for regressions with the predictor “Sum of Operands” [*t*(18) = 2.72, *p* = 0.014], as well as for regressions with the predictor “Product of Operands” [*t*(18) = 4.66, *p* < 0.0001]. No significant difference between strategy ratings and predictors of number size was observed in regressions on reaction times of all tasks, as well as in regressions on reaction times of multiplication tasks. However, in both cases strategy ratings and number size predictors explained a substantial amount of independent variance, suggesting the importance of strategy ratings as measure of task complexity in all conducted regression analyses.

### Receiver Operating Characteristic Analysis

We used a ROC analysis to determine whether reaction times differentiate better between strategy ratings (retrieval vs. multi-step procedural) or between task features (tasks involving 1-digit vs. 2-digit numbers). The analyzed AUC value provides the likelihood that, given a true positive and a true negative, the ROC analysis correctly classifies the variable “Ratings” or “Task Features.” In the case of ratings, true positives would refer to the percentage of retrieval trials that were accurately identified as retrieval trials, and false positives would refer to the percentage of procedural trials that were inaccurately identified as retrieval trials. An AUC of 1 indicates perfectly accurate classification.

**FIGURE 5:**
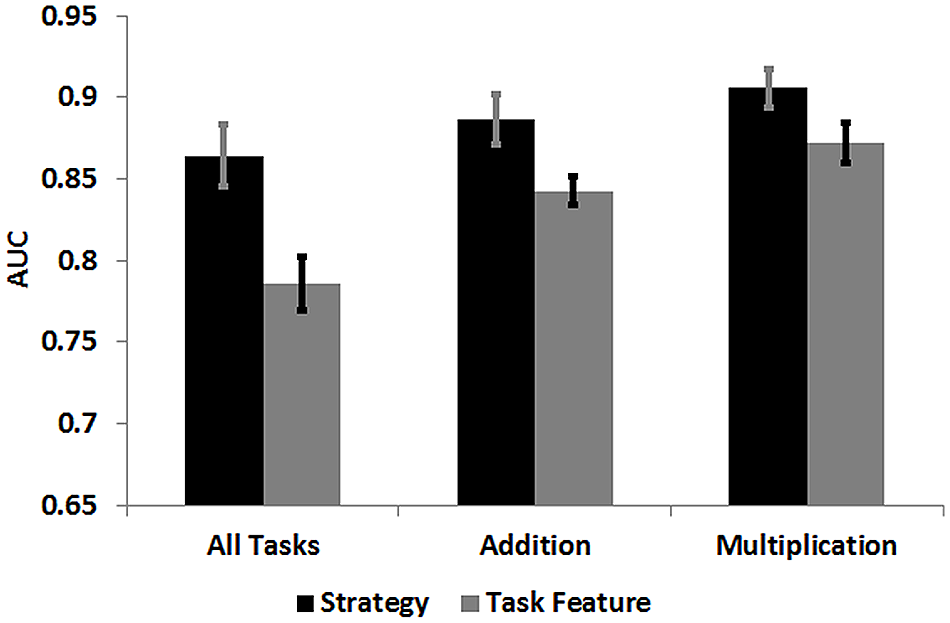
**Mean Area Under Curve (AUC) values from ROC analyses on all tasks, as well as addition and multiplication tasks.** Error bars indicate the SE of the mean.

Receiver Operating Characteristic analyses revealed a significant classification difference of reaction times for strategies and task features. For analyses of all tasks, a significantly higher AUC value was observed for strategies (mean AUC value = 0.86) than for task features [mean AUC value = 0.78; *t*(18) = 4.49, *p* < 0.0001; **Figure 5**, **Table 4**]. This was also the case for analyses on addition tasks, which revealed a significantly higher AUC value for strategies (mean AUC value = 0.88) than for task features [mean AUC value = 0.84; *t*(18) = 2.38, *p* = 0.028]. For analyses on multiplication tasks, a marginal significant differences between strategies (mean AUC value = 0.90) and task features (mean AUC value = 0.87) was observed [*t*(18) = 1.96, *p* = 0.065] due to the fact that reaction times also classified task features very well. Overall, the current ROC analysis provides strong evidence that reaction times differentiate types of arithmetic strategies well, and that they classify strategy ratings significantly better than task features in analyses including all tasks and addition tasks.

**Table 4:** Mean and SD of AUC values from ROC analyses of all arithmetic tasks, as well as addition and multiplication tasks.

		Area under the Curve (AUC) values				
	**All arithmetic tasks**		**Addition**		**Multiplication**	
	**Strategy**	**Task features**	**Strategy**	**Task features**	**Strategy**	**Task features**

Mean	0.86	0.78	0.88	0.84	0.90	0.87
SD	0.08	0.07	0.06	0.03	0.05	0.05

## Discussion

In the current study we evaluated different measures of task complexity in an arithmetic task paradigm that is suitable for future neuroimaging research. The relative predictive value of self-reported strategies for arithmetic performance was analyzed and compared with objective task complexity criteria based on number size.

Overall, strategy ratings outperformed task features in most of our analyses, suggesting that strategy assessment in our neuroimaging research compatible paradigm is a powerful measure of problem complexity across different task types, and should be incorporated in future neuroimaging research on arithmetic problem solving. This is in line with evidence from two recent EEG studies showing that trial-by-trial self-reported strategies explain neural effects better than complexity measures based on number size (Grabner and De Smedt, 2011, 2012), as well as evidence from an fMRI study in which self-reported strategies yielded a better match in task complexity across arithmetic conditions, compared to number size based complexity criteria (Tschentscher and Hauk, 2014). Therefore, we propose that neuroimaging studies that aim to compare different types of tasks (for example arithmetic operation types), should match these tasks with respect to individual ratings of task difficulty.

### Strategy Ratings are the most Powerful Predictor of Task Complexity

In the PCA, over 90 percent of variance in reaction times of addition and multiplication tasks was explained by the first component. High factor loadings of this first component were observed for task categories that varied in number sizes between combinations of 1/1-digit numbers and 2/2-digits numbers. Hence, number size, which has been a common criterion of task complexity in many previous neuroimaging studies, did not reflect component loadings well. When comparing patterns of strategy ratings and mean reaction times with PCA results, it is striking that the pattern of strategy choices closely matched the factor loadings of the first PCA component (**Figure 3**). This finding gains further support by results of simultaneous multiple regression analyses. Strategy ratings explained about one third more variance in reaction times than number size predictors. This difference was more pronounced in addition tasks, while strategy and number size predictors explained substantial amounts of independent variance in all analyses. This suggests that strategy ratings should be considered as additional criterion for complexity beyond task features in future studies. We finally assessed whether a ROC curve more appropriately classifies self-reported strategies (direct retrieval vs. multi-step procedural) or number size based complexity levels (tasks involving 1-digit numbers vs. 2-digit numbers), and could provide evidence for the superiority of strategy ratings. The classification accuracy of strategy ratings across all tasks was high (mean AUC = 0.86), and significantly differed from classifications based on number size (mean AUC = 0.78).

### Limitations of Strategy Reports

While strategy ratings were the best predictor of task performance in the current study, they may still be affected by response biases in a different experimental setting. The current group of participants was relatively high performing, with average and above average IQ. However, the predictive value of strategy ratings might differ for groups of participants with lower overall skills. For example, it has been shown that the performance of participants with low arithmetic fluency is more affected by trial-by-trial strategy reports in both error rates and reaction times, than the performance of higher-skilled participants (Smith-Chant and LeFevre, 2003). Hence, performance differences between groups may be enhanced when strategy measures are simultaneously obtained. Further, strategy reports might be biased by the way experimental instructions are provided, resulting in systematic biases toward more or less reports of direct memory retrieval (Kirk and Ashcraft, 2001). This can be a concern when comparing proportions of partial strategy choices (memory retrieval vs. procedural) across studies.

## Conclusion

The ideal measure for task complexity would be based on detailed knowledge of the cognitive and neural mechanisms contributing to the problem solving process, and how they are affected by individual differences. While a number of theoretical and neuroscientific proposals for the mechanisms of problem solving exist (Baddeley, 1986; Anderson, 2005; Duncan, 2010), a generally applicable model for task complexity has not been established yet. The current study aims to provide a pragmatic approach toward better control of task complexity effects in neuroimaging research. Many studies – such as neuroimaging studies on neural differences between arithmetic operation types – do not explicitly address questions about task complexity or individual participants’ arithmetic strategies. However, these studies have to rule out that differences in task complexity across conditions, i.e., differences in the use of arithmetic strategies, confound their hypothesized specific neural differences between arithmetic operation types. If only reaction times were considered, it would not be clear how to select trials in order to match stimulus conditions for complexity. Our rating procedure offers a principled way to categorize stimulus categories in a factorial design, as used in most neuroimaging studies on numerical cognition. We recommend individual strategy ratings as a pragmatic measure for task complexity, possibly in combination with reaction time assessment and objective task-based measures, especially for cases where detailed cognitive models of task complexity are not available.

Our conclusion that individual strategy ratings capture task complexity better than objective task features is based on our finding that individual ratings were the most reliable predictor for behavioral performance across different analyses conducted in the current study. The relevance of this finding has already been indicated by a few neuroimaging studies (Grabner et al., 2009; Grabner and De Smedt, 2011, 2012; Tschentscher and Hauk, 2014), where classification of task complexity via arithmetic strategies significantly changed the results. However, according to our knowledge we here provide first evidence on the superiority of strategy ratings based on the direct comparison between subjective ratings and objective task complexity criteria.

## Conflict of Interest Statement

The authors declare that the research was conducted in the absence of any commercial or financial relationships that could be construed as a potential conflict of interest.

## References

[B1] Anderson J. R. (2005). Human symbol manipulation within an integrated cognitive architecture. *Cogn. Sci.* 29 313–341. 10.1207/s15516709cog0000_22 21702777

[B2] Arsalidou M. Taylor M. J. (2011). Is 2+2=4? Meta-analyses of brain areas needed for numbers and calculations. *Neuroimage* 54 2382–2393. 10.1016/j.neuroimage.2010.10.009 20946958

[B3] Baddeley A. D. (1986). *Working Memory.* Oxford: Clarendon Press.

[B4] Badets A. Pesenti M. Olivier E. (2010). Response-effect compatibility of finger-numeral configurations in arithmetical context. *Q. J. Exp. Psychol.* 63 16–22. 10.1080/17470210903134385 19691005

[B5] Baroody A. J. Dowker A. (2003). *The Development of Arithmetic Concepts and Skills: Constructing Adaptive Expertise.* Mahwah, NJ: Lawrence Erlbaum Associates.

[B6] Barrouillet P. Thevenot C. (2013). On the problem-size effect in small additions: can we really discard any counting-based account? *Cognition* 128 35–44. 10.1016/j.cognition.2013.02.018 23583543

[B7] Campbell J. I. D. Penner-Wilger M. (2006). Calculation latency: the mu of memory and the tau of transformation. *Mem. Cogn.* 34 217–226. 10.3758/BF03193400 16686120

[B8] Cattell R. B. Cattell A. K. S. (1960). *Handbook for the Individual or Group Culture Fair Intelligence Test.* Champaign, IL: Testing Inc.

[B9] Centor R. M. (1991). Signal Detectability: the Use of ROC Curves and Their Analyses. *Med. Decis. Making* 11 102–106. 10.1177/0272989X9101100205 1865776

[B10] Chochon F. Cohen L. van de Moortele P. F. Dehaene S. (1999). Differential contributions of the left and right inferior parietal lobules to number processing. *J. Cogn. Neurosci.* 11 617–630. 10.1162/089892999563689 10601743

[B11] De Smedt B. Grabner R. H. Studer B. (2009). Oscillatory EEG correlates of arithmetic strategy use in addition and subtraction. *Exp. Brain Res.* 195 635–642. 10.1007/s00221-009-1839-9 19452143

[B12] Duncan J. (2010). The multiple-demand (MD) system of the primate brain: mental programs for intelligent behaviour. *Trends Cogn. Sci.* (*Regul. Ed.*) 14 172–179. 10.1016/j.tics.2010.01.004 20171926

[B13] Fawcett T. (2006). An introduction to ROC analysis. *Pattern Recognit. Lett.* 27 861–874. 10.1016/j.patrec.2005.10.010

[B14] Fehr T. Code C. Herrmann M. (2007). Common brain regions underlying different arithmetic operations as revealed by conjunct fMRI-BOLD activation. *Brain Res.* 1172 93–102. 10.1016/j.brainres.2007.07.043 17822681

[B15] Fischer M. H. (2012). A hierarchical view of grounded, embodied, and situated numerical cognition. *Cogn. Process.* 13 161–164. 10.1007/s10339-012-0477-5 22802036

[B16] Grabner R. H. Ansari D. Koschutnig K. Reishofer G. Ebner F. Neuper C. (2009). To retrieve or to calculate? Left angular gyrus mediates the retrieval of arithmetic facts during problem solving. *Neuropsychologia* 47 604–608. 10.1016/j.neuropsychologia.2008.10.013 19007800

[B17] Grabner R. H. De Smedt B. (2011). Neurophysiological evidence for the validity of verbal strategy reports in mental arithmetic. *Biol. Psychol.* 87 128–136. 10.1016/j.biopsycho.2011.02.019 21382434

[B18] Grabner R. H. De Smedt B. (2012). Oscillatory EEG correlates of arithmetic strategies: a training study. *Front. Psychol.* 3:428 10.3389/fpsyg.2012.00428 PMC3498901 23162495

[B19] Hauk O. Tschentscher N. (2013). The body of evidence: what can neuroscience tell us about embodied semantics? *Front. Cogn. Sci.* 4:50 10.3389/fpsyg.2013.00050 PMC3570773 23407791

[B20] Hecht S. A. (2006). Group differences in adult simple arithmetic: good retrievers, not-so-good retrievers, and perfectionists. *Mem. Cogn.* 34 207–216. 10.3758/BF03193399 16686119

[B21] Imbo I. Vandierendonck A. Rosseel Y. (2007). The influence of problem features and individual differences on strategic performance in simple arithmetic. *Mem. Cogn.* 35 454–463. 10.3758/BF03193285 17691144

[B22] Jost K. Beinhoff U. Hennighausen E. Rosler F. (2004). Facts, rules, and strategies in single-digit multiplication: evidence from event-related brain potentials. *Cogn. Brain Res.* 20 183–193. 10.1016/j.cogbrainres.2004.02.005 15183390

[B23] Jost K. Khader P. Burke M. Bien S. Rosler F. (2009). Dissociating the solution processes of small, large, and zero multiplications by means of fMRI. *Neuroimage* 46 308–318. 10.1016/j.neuroimage.2009.01.044 19457376

[B24] Kirk E. P. Ashcraft M. H. (2001). Telling stories: the perils and promise of using verbal reports to study math strategies. *J. Exp. Psychol. Learn. Mem. Cogn.* 27 157–175. 10.1037/0278-7393.27.1.157 11204096

[B25] Klein E. Moeller K. Willmes K. Nuerk H.-C. Domahs F. (2011). The influence of implicit hand-based representations on mental arithmetic. *Front. Psychol.* 2:197 10.3389/fpsyg.2011.00197 PMC3169791 21927606

[B26] Kong J. Wang C. Kwong K. Vangel M. Chua E. Gollub R. (2005). The neural substrate of arithmetic operations and procedure complexity. *Cogn. Brain Res.* 22 397–405. 10.1016/j.cogbrainres.2004.09.011 15722210

[B27] Lakoff G. Núñez R. E. (2000). *Where Mathematics Comes from: How the Embodied Mind Brings Mathematics into Being.* New York: Basic Books.

[B28] LeFevre J. A. Bisanz J. Daley K. E. Buffone L. Greenham S. L. Sadesky G. S. (1996a). Multiple routes to solution of single-digit multiplication problems. *J. Exp. Psychol. Gen.* 125 284–306. 10.1037/0096-3445.125.3.284

[B29] LeFevre J. A. Sadesky G. S. Bisanz J. (1996b). Selection of procedures in mental addition: reassessing the problem size effect in adults. *J. Exp. Psychol. Learn. Mem. Cogn.* 22 216–230. 10.1037/0278-7393.22.1.216

[B30] LeFevre J. A. DeStefano D. Penner-Wilger M. Daley K. E. (2006). Selection of procedures in mental subtraction. *Can. J. Exp. Psychol.* 60 209–220. 10.1037/cjep2006020 17076436

[B31] LeFevre J. A. Smith-Chant B. L. Hiscock K. Daley K. E. Morris J. (2003). “Young adults’ strategic choices in simple arithmetic: implications for the development of mathematical representations,” In *The Development of Arithmetic Concepts and Skills: Recent Research and Theory*, eds Baroody A. J. Dowker A. (Hillsdale, NJ: Erlbaum), 203–228.

[B32] Lemaire P. Arnaud L. (2008). Young and older adults’ strategies in complex arithmetic. *Am. J. Psychol.* 121 1–16. 10.2307/20445440 18437798

[B33] Lilliefors H. W. (1969). On the Kolmogorov-Smirnov Test for the exponential distribution with mean unknown. *J. Am. Stat. Assoc.* 64 387–389. 10.1080/01621459.1969.10500983

[B34] Mazzocco M. M. M. Thompson R. E. (2005). Kindergarten predictors of math learning disability. *Learn. Disabil. Res. Pract.* 20 142–155. 10.1111/j.1540-5826.2005.00129.x 20084182 PMC2806680

[B35] Menon V. Rivera S. M. White C. D. Glover G. H. Reiss A. L. (2000). Dissociating prefrontal and parietal cortex activation during arithmetic processing. *Neuroimage* 12 357–365. 10.1006/nimg.2000.0613 10988030

[B36] Rosenberg-Lee M. Chang T. T. Young C. B. Wu S. Menon V. (2011). Functional dissociations between four basic arithmetic operations in the human posterior parietal cortex: a cytoarchitectonic mapping study. *Neuropsychologia* 49 2592–2608. 10.1016/j.neuropsychologia.2011.04.035 21616086 PMC3165023

[B37] Rosenberg-Lee M. Lovett M. C. Anderson J. R. (2009). Neural correlates of arithmetic calculation strategies. *Cogn. Affect. Behav. Neurosci.* 9 270–285. 10.3758/CABN.9.3.270 19679763

[B38] Smith-Chant B. L. LeFevre J. A. (2003). Doing as they are told and telling it like it is: Self-reports in mental arithmetic. *Mem. Cogn.* 31 516–528. 10.3758/BF03196093 12872868

[B39] Tschentscher N. Hauk O. (2014). How are things adding up? Neural differences between arithmetic operations are due to general problem solving strategies. *Neuroimage* 92 369–380. 10.1016/j.neuroimage.2014.01.061 24525170

[B40] Wu S. S. Meyer M. L. Maeda U. Salimpoor V. Tomiyama S. Geary D. C. (2008). Standardized assessment of strategy use and working memory in early mental arithmetic performance. *Dev. Neuropsychol.* 33 365–393. 10.1080/87565640801982445 18473204 PMC3607623

[B41] Zhou X. L. Chen C. S. Dong Q. Zhang H. C. Zhou R. L. Zhao H. (2006). Event-related potentials of single-digit addition, subtraction, and multiplication. *Neuropsychologia* 44 2500–2507. 10.1016/j.neuropsychologia.2006.04.003 16828126

[B42] Zhou X. L. Chen C. S. Zang Y. F. Dong Q. Chen C. H. Qiao S. B. (2007). Dissociated brain organization for single-digit addition and multiplication. *Neuroimage* 35 871–880. 10.1016/j.neuroimage.2006.12.017 17292628

